# Characterization of two *exoU*^+^*/exoS*^+^ carbapenem-non-susceptible *Pseudomonas aeruginosa* co-colonizing the lung of a bacterial pneumonia patient

**DOI:** 10.1186/s12866-026-05172-8

**Published:** 2026-05-18

**Authors:** Yi Yan, Lvxin Qian, Xiaoqin Feng, Furong Zhang, Min Tang, Huan Chen, Ying Li, Luhua Zhang

**Affiliations:** 1 The School of Basic Medical Sciences, Southwest Medical University, Luzhou, Sichuan China; 2 Department of Clinical Laboratory, Pengzhou People’s Hospital, Chengdu, Sichuan China; 3 Department of Clinical Laboratory, The Affiliated Hospital of Southwest Medical University, Luzhou, Sichuan China

**Keywords:** *Pseudomonas aeruginosa*, Carbapenem-non-susceptible, *exoU*, Hypermutator, High virulence

## Abstract

**Background:**

*Pseudomonas aeruginosa* is a major cause of acute nosocomial infections, as well as chronic respiratory infections associated with cystic fibrosis (CF). In chronic lung infections, *P. aeruginosa* populations typically exhibit extensive phenotypic variation, a trait linked to their need to undergo pathoadaptive mutations to counteract host-derived selective pressures.

**Methods:**

In this study, two clonally related *P. aeruginosa* isolates, SCPA07 and SCPA08, were identified to coexist in a single bronchoalveolar lavage fluid (BALF) sample from a patient with bacterial pneumonia. Whole-genome sequencing (WGS) was conducted to characterize their genetic background, as well as antimicrobial resistance and virulence gene profiles. A comprehensive analysis of phylogenetic relationships and comparative genomic features of the two isolates was conducted using a panel of bioinformatics tools. Their antimicrobial resistance mechanisms were elucidated via gene sequence analysis and quantitative reverse-transcription PCR (qRT-PCR). A series of phenotypic experiments, including growth, biofilm formation, environmental stress, and virulence assays, and others were performed to characterize their phenotypic traits.

**Results:**

Antimicrobial susceptibility assay showed that both strains were carbapenem-non-susceptible (defined as intermediate or resistant according to the Clinical and Laboratory Standards Institute (CLSI) guidelines). Genomic analysis revealed that they are ‘hypermutator’ strains and harbor both the *exoS* and *exoU* virulence genes, indicating an increased propensity for persistent host infection and high virulence potential. Both strains harbored mutations in *oprD*, and exhibited elevated expression of AmpC β-lactamase and the efflux pump MexB, which most likely contributed to their non-susceptibility to carbapenems. Despite harboring nucleotide variations at only ten genetic loci, these two strains exhibited distinct phenotypic traits: SCPA07 showed rapid growth, strong biofilm formation, high virulence, and growth advantages under iron limitation and serum stress; in contrast, its variant SCPA08 had slow growth, poor motility, reduced pyocyanin production, low virulence, and increased tolerance to the host antimicrobials human cathelicidin LL-37 and hydrogen peroxide (H_2_O_2_). These phenotypic variations are proposed to be primarily driven by genetic mutations affecting the O-antigen biosynthesis, iron utilization, Porin D, and other determinants.

**Conclusions:**

This study elucidates the divergent adaptive evolutionary strategies of a single *exoS*^+^/*exoU*^+^ *P. aeruginosa* clone within the host during bacterial pneumonia, as well as their critical role in shaping the bacterium’s virulence and adaptability, which sheds light on the within-host evolution dynamics of *P. aeruginosa* populations during their pathogenesis and persistence in the lung.

**Supplementary Information:**

The online version contains supplementary material available at 10.1186/s12866-026-05172-8.

## Background

*Pseudomonas aeruginosa*, a ubiquitous Gram-negative environmental bacterium, is an opportunistic human pathogen that causes various life-threatening acute and chronic infections in immunocompromised patients [1]. Driven by its vast array of virulence factors and combined intrinsic and acquired antibiotic resistance mechanisms, *P. aeruginosa* is one of the leading nosocomial pathogens and the primary cause of morbidity and mortality in cystic fibrosis patients [2]. 2022 China Antimicrobial Surveillance Network surveillance data revealed that *P. aeruginosa* was the 4th most frequently isolated pathogen among all clinical isolates in China, with carbapenem-resistant *P. aeruginosa* at an average prevalence of 23.8% [3]. In *P. aeruginosa*, carbapenem resistance is most commonly mediated by the acquisition of carbapenem-hydrolyzing β-lactamases, reduced permeability of outer membrane porin OprD, chromosomal AmpC overproduction, and enhanced efflux pump expression [4]. The virulence of *P. aeruginosa* is closely linked to the type III secretion system, which injects cytotoxins, including ExoU, ExoT, ExoS, and ExoY, to severely impair host defense [5]. Among them, ExoS and ExoU are the most efficiently translocated effectors and major contributors to pathogenesis, with cytotoxic isolates typically secreting ExoU and invasive isolates secreting ExoS [6]. Previous studies have demonstrated that the large majority of *P. aeruginosa* isolates contained either the *exoU* or *exoS* gene; for example, reference strain PA14 only expresses the *exoU* gene, while strain PAO1 only expresses the *exoS* gene [7]. Song et al. demonstrated that those *P. aeruginosa* strains co-carrying *exoU* and *exoS* genes exhibited hypervirulence when compared to PAO1 (*exoS*^*+*^*/exoU*^*−*^) and PA14 (*exoS*^−^/*exoU*^+^) [8]. Zhu et al. reported an emergence of *exoS*^+^/*exoU*^+^, imipenem-nonsusceptible ST463 *P. aeruginosa* isolates in Zhejiang province, southeast China, and most of them exhibit multidrug resistance and hypervirulence [9]. Infections caused by these hypervirulent and carbapenem-non-susceptible strains would pose substantial therapeutic challenges and economic burdens.

Treatments of *P. aeruginosa* infections are extremely difficult due to its rapid mutations and adaptive evolution, which enable the bacterium to develop robust resilience to environmental changes and thus enhance its survival in the host [10]. The ecological flexibility of *P. aeruginosa* can be attributed to its relatively large genome (5.5–7 Mb), which encodes a high proportion of regulatory genes, as well as catabolic elements, transporters, and virulence factors [11]. To achieve long-term adaptation and survival within the host, *P. aeruginosa* develops a series of phenotypic changes via the accumulation of pathoadaptive mutations [10]. Due to defects in DNA mismatch repair systems, the clinical populations of *P. aeruginosa* designated as ‘hypermutators’ consequently display elevated mutation rates, which further enhance the potential for these bacterial pathogens to genetically adapt to the host immune system and drug therapies [12]. Some common adaptive phenotypic traits are selected for in genetically distinct *P. aeruginosa* isolates from chronically infected CF patients, such as slow growth, impaired motility, loss of quorum sensing, increased biofilm formation, and overproduction of alginate [13], indicating parallel evolution of these strains within the hosts.

In spite of the parallelism in the phenotypic changes among clinical isolates, there is extensive phenotypic diversity within populations of *P. aeruginosa* in an individual patient, indicative of multiple evolutionary trajectories toward pathoadaptation [10]. In this study, two carbapenem-non-susceptible *P. aeruginosa* strains with distinct colony sizes were isolated from the same BALF sample of a patient with bacterial pneumonia. Genomic analysis revealed these two strains are clonally related ‘hypermutator’ strains, and they co-harbor the *exoU* and *exoS* virulence genes. We further correlated the genetic variations with their corresponding phenotypic alterations of each strain, respectively, so as to elucidate the divergent adaptive evolutionary strategies of these two clonal strains within the lung microenvironment.

## Methods

### Bacterial strains and growth conditions

The SCPA07 and SCPA08 strains used in this study were isolated from a BALF sample of a patient with bacterial pneumonia. The strains were identified as *P. aeruginosa* with the AutoFMS600 system using MALDI-TOF MS technology (Autobio Diagnostics CO., Ltd, China). Unless otherwise specified, strains were routinely grown in Luria-Bertani (LB) broth at 37 °C with shaking at 220 rpm overnight to obtain logarithmic-phase cultures. Alternatively, strains were streaked onto LB agar plates and incubated statically at 37 °C overnight. When required, streptomycin or tetracycline was supplemented into the medium at a final concentration of 10 µg/mL.

### Congo red colony morphology assay

For the analysis of colony morphology on Congo red agar, 1.8 µL of bacterial overnight culture was spot-inoculated onto LB agar plates supplemented with 40 µg/mL Congo red and 1 µg/mL Coomassie brilliant blue [14]. The plates were incubated at 30 °C overnight and then statically placed at room temperature for an additional 24 h for colony morphology observation.

### WGS and genomic analyses

WGS was performed for clinical isolates SCPA07 and SCPA08 to characterize their genomic features. Both strains were subjected to 150‑bp paired‑end sequencing on the Illumina NovaSeq 6000 platform at a sequencing depth of ~ 200×. Raw reads were trimmed using Trimmomatic v0.38 [15] and then assembled into draft genomes using SPAdes v3.12.0 [16]. To obtain a complete genome, SCPA07 was additionally sequenced using a hybrid approach combining Nanopore PromethION long-read and Illumina NovaSeq PE150 short-read technologies, followed by hybrid assembly with Unicycler v0.4.3 [17]. Genome annotation was performed using Prokka v1.14 [18]. Average nucleotide identity (ANI) was calculated using the ANI Calculator [19]. Prediction of antibiotic resistance genes (ARGs) and virulence genes was performed using the ResFinder database [20] and Virulence Factor Database (VFDB), respectively [21]. Single-nucleotide polymorphism (SNP) analysis was performed by aligning the genome sequence of SCPA08 with that of SCPA07 using Breseq v0.39.0 [22]. Sequence alignments of key genes were performed in SnapGene (GSL Biotech, http://www.snapgene.com/).

### Phylogenetic analysis

Phylogenetic analysis of SCPA07, SCPA08, and other representative *P. aeruginosa* strains was conducted based on core-genome SNPs as previously described [23]. Briefly, genomic annotation of all isolates was performed using Prokka v1.14, and the resulting GFF3 files were used as input for Roary to generate a core genome alignment [24]. Core genome SNPs were extracted from the alignment using snp-sites v2.3.2 [25]. Based on the identified core-genome SNPs, a maximum-likelihood phylogenetic tree was constructed using FastTree v2.1.10 with the GTR model [26]. Node support was assessed by 1,000 bootstrap replicates (values ≥ 0.9 shown).

### Growth curve measurement, biofilm detection

Bacterial growth was monitored via microplate assay. Overnight cultures of strains SCPA07 and SCPA08 were adjusted to an OD₆₀₀ of 0.1 with fresh LB medium and further diluted 1:100. A 200 µL aliquot of each dilution was transferred to a 96-well plate (Corning, USA), and the plate was incubated at 37 °C for 24 h in a microplate reader (BioTek, Synergy H1). OD₆₀₀ was measured every 60 min after automatic shaking, and growth curves were plotted from the OD data. The area under the curve (AUC) was calculated using GraphPad Prism 10.1.2. All experiments were conducted with three independent biological replicates.

Biofilm formation was quantified in 96-well plates using crystal violet staining [27]. Overnight cultures were diluted 1:100 in LB broth and incubated statically at 37 °C for 24 h. The initial inoculum was determined via serial dilution and colony-forming units (CFU) counting. After removing planktonic cells, adherent biofilms were washed, methanol-fixed, stained with 0.1% crystal violet, and washed again. The bound dye was solubilized with 33% acetic acid, and absorbance was measured at 595 nm. Biofilm formation was expressed as OD₅₉₅ normalized to the corresponding initial CFU count.

### Measurement of pyocyanin production

Pyocyanin was extracted and quantified following a previously described method with minor modifications [28]. Overnight bacterial cultures were centrifuged at 12,000 rpm for 2 min. The supernatant (5 mL) was extracted with 3 mL of chloroform. Following phase separation, the chloroform layer was collected and vigorously mixed with 1 mL of 0.2 M HCl. After centrifugation, the absorbance of the resulting aqueous phase was measured at 520 nm. Pyocyanin concentration was calculated as (A₅₂₀ × 17.072) / OD₆₀₀ and expressed in µg/mL/OD₆₀₀.

### Swimming and swarming assay

Motility assays were performed as described [14]. The swimming medium contained 0.3% agar, 1% tryptone, and 0.5% NaCl, and the swarming medium contained 0.5% agar, 0.8% nutrient broth, and 0.5% glucose. Overnight cultures in LB broth were adjusted to an OD₆₀₀ of 1.0, and 2 µL aliquots were spot-inoculated onto the center of the respective agar plates. Plates were incubated at 30 °C (swimming) or 37 °C (swarming) for 14–16 h. Motility was quantified by measuring the diameter of the bacterial migration zone.

### Pyoverdine production

Bacterial cultures were grown in 3 mL of King’s B medium at 37 °C with shaking (220 rpm) for 48 h. After measuring the OD₆₀₀, cultures were centrifuged, and the supernatant was collected for pyoverdine quantification [29]. Fluorescence was measured (excitation 400 nm, emission 460 nm) using a Synergy H1 microplate reader (BioTek). Pyoverdine production was expressed as relative fluorescence units (RFU) normalized to culture density (RFU/OD₆₀₀). The experiment was performed with three independent biological replicates, each in technical triplicate.

### Iron-limited growth assay

The iron-restricted spotting assay was performed as previously described with minor modifications [30]. Briefly, *P. aeruginosa* strains were grown to logarithmic phase in LB broth and adjusted to 10^8^ CFU/mL. Serial dilutions in PBS were prepared, and 5 µL aliquots of each dilution were spotted onto LB agar plates supplemented with 300 µM 2,2′-dipyridyl. Plates were incubated at 37 °C for 12 h, and growth was assessed.

### Siderophore secretion assay

Siderophore production was evaluated using chrome azurol S (CAS)-LB agar plates [31]. A 10× stock solution of CAS was prepared by dissolving 6.04 mg CAS, 7.29 mg cetyltrimethylammonium bromide (CTAB), and 1.35 mg FeCl_3_·6H_2_O in 10 mL deionized water, followed by filter sterilization. Blue CAS-LB agar plates were prepared by mixing 10 mL of this stock solution with 90 mL of autoclaved LB agar. Overnight M9 cultures (10 µL) were spotted onto plates and incubated at 37 °C for 48 h. The diameter of the resulting orange-yellow halo was measured with a caliper.

### Serum bactericidal assay

Serum sensitivity was assessed as described previously with minor modifications [32]. *P. aeruginosa* strains were grown to logarithmic phase in LB broth, harvested by centrifugation (6,000 × g, 5 min), and resuspended in PBS to a final OD₆₀₀ of 0.1. The bacterial suspension was diluted 1:10 in PBS, and 10 µL was mixed with 40 µL of fresh serum, followed by incubation at 37 °C. At 0, 1, and 3 h, samples were serially diluted and plated to determine viable counts (CFU/mL). PBS control was included at each time point. The survival rate was calculated as (CFU in serum / CFU in PBS control) × 100%. Experiments were performed with three biological replicates, each in technical triplicate.

### H_2_O_2_ sensitivity assay

Bacterial susceptibility to hydrogen peroxide was evaluated using an established method with modifications [33]. Logarithmic-phase bacterial cultures were harvested by centrifugation, resuspended in 50 mM H_2_O_2_, and incubated at 37 °C with shaking for 20 min. Cells were then washed, serially diluted in sterile water, plated onto LB agar, and incubated overnight at 37 °C for CFU enumeration. Bacteria treated with sterile water were used as the control. The experiment was performed with three biological replicates, each in technical triplicate.

### LL-37 sensitivity assay

The antimicrobial activity of LL-37 (Cayman Chemical, USA) was assessed against exponential-phase *P. aeruginosa* strains as previously described [34]. Bacterial suspensions in PBS were mixed 1:1 (v/v) with LL-37 at a final concentration of 50 µg/mL and incubated at 37 °C for 1 h. After incubation, samples were serially diluted, plated on LB agar, and incubated overnight at 37 °C for CFU enumeration. A PBS-only control was included in parallel. The assay was performed with three biological replicates, each in technical triplicate.

### *Galleria mellonella (G. mellonella)* killing assays

Healthy *G. mellonella* larvae (200–350 mg) were selected for this experiment. A single colony from an overnight LB agar plate was inoculated into LB broth and grown to mid-log phase at 37 °C with shaking. Cells were harvested, washed, and adjusted to OD₆₀₀ ≈ 0.1 (~ 1 × 10^8^ CFU/mL) in PBS. Ten-fold serial dilutions yielded the target infection dose of ~ 5 × 10^5^ CFU/mL, verified by retrospective plating. A 10 µL aliquot of bacterial suspension was injected into the last pair of abdominal prolegs of each larva (*n* = 10 per strain) [28]. Larvae inoculated with 10 µL of sterile PBS served as the control. Injected larvae were incubated at 37 °C, and survival was monitored every 6 h. Larvae unresponsive to gentle touch were recorded as dead.

### qRT-PCR

*P. aeruginosa* strains were cultured to mid-log phase for qRT-PCR assay as previously described [35]. Total RNA was extracted using the MiniBEST Universal RNA Extraction Kit (TaKaRa, Japan), and reverse transcription was performed with the PrimeScrip™ RT Reagent Kit (with gDNA Eraser, TaKaRa) according to the manufacturer’s instructions.

qRT-PCR was conducted on a LineGene 9600 Plus system (BIOER Technology) under the following conditions: initial denaturation at 95 °C for 30 s; 40 cycles of 95 °C for 10 s, 63 °C for 30 s, and 72 °C for 30 s; followed by melting curve analysis. Primer sequences are listed in Supplementary Table 3. The housekeeping gene *rpsL* was used for normalization, and relative expression levels were calculated using the 2^–ΔΔCt^ method. Experiments included three biological replicates, each with three technical replicates.

### Statistics and reproducibility

Statistical analyses were performed using GraphPad Prism 10.1.2 (GraphPad Software, La Jolla, CA, USA). Data from at least three independent biological replicates are expressed as mean ± SD. One-way ANOVA with Tukey’s post hoc test was applied in the following assays: bacterial growth, biofilm formation, pyocyanin and pyoverdine production, survival ratio under H_2_O_2_, LL-37, and serum challenge, and gene expression by qRT-PCR. Survival curves from *G. mellonella* infection were analyzed using the log-rank (Mantel–Cox) test. Significance levels are denoted as *P < 0.05, **P < 0.01, ***P < 0.001, and ****P < 0.0001.

## Results

### Concurrent isolation of two carbapenem-non-susceptible *P. aeruginosa* strains from the same sample

**Fig. 1:**
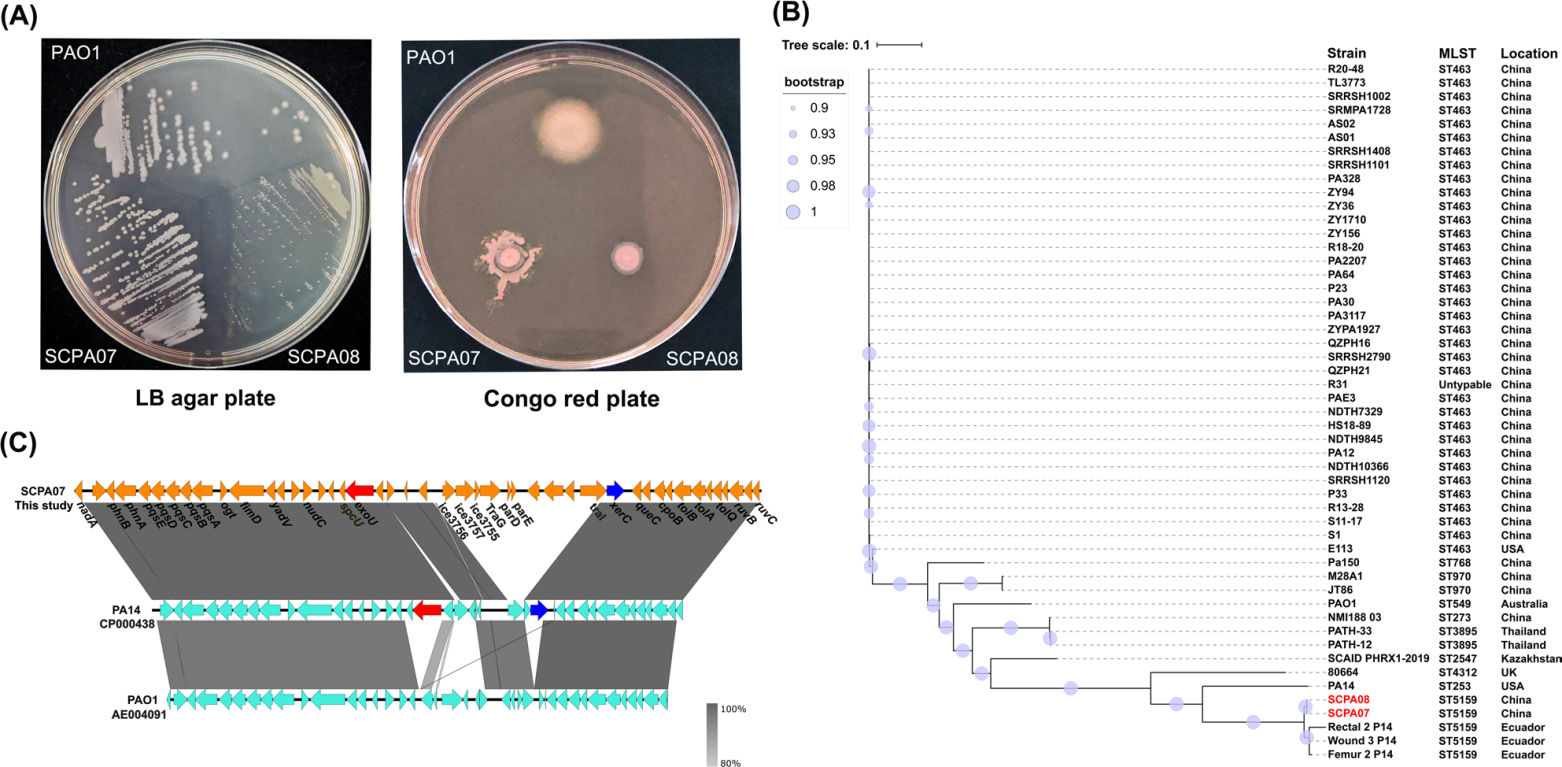
Characterization of *P. aeruginosa* clinical isolates SCPA07 and SCPA08. **A **Colony morphology of SCPA07 and SCPA08 after 24 h of incubation at 37 °C on LB agar plates and Congo red agar plates. **B** Phylogenetic tree constructed based on core genome SNPs, including SCPA07 and SCPA08, reference strains PAO1 and PA14, and 42 *exoS*^*+*^*/exoU*^*+*^ *P. aeruginosa* strains with complete genomes retrieved from the NCBI database. **C** Comparative genomics of the PAPI-2 virulence island. Schematic alignment of the *exoU*-containing PAPI-2 region among *P. aeruginosa* strains SCPA07 (this study), PA14, and PAO1. Genes are depicted as arrows, with the virulence factor gene *exoU* highlighted in red and the recombination gene *xerC* in blue. Nucleotide sequence homology (80–100%) between SCPA07 and the reference strains is indicated by gray shading (see gradient bar)

Two clinical *P. aeruginosa* strains, SCPA07 and SCPA08, were isolated from the same BALF sample from the 69-year-old male patient diagnosed with bacterial pneumonia on December 29, 2023. After 24 h of incubation at 37 °C on LB solid medium (Fig. 1A), SCPA07 produced large colonies (2.0–3.0 mm in diameter) that were highly similar in morphology to the reference strain PAO1. These colonies were round, with well-demarcated and smooth moist surfaces. Notably, SCPA07 secreted a distinct diffusible reddish pigment that encircled the colonies. By contrast, SCPA08 formed smaller colonies (0.5–1.0 mm in diameter) with a compact, round shape, distinct margins, and smooth, moist surfaces, and did not produce any visible diffusible pigments. Both SCPA07 and SCPA08 were defined as non-mucoid strains.

Antimicrobial susceptibility testing revealed that both strains were resistant to ticarcillin/clavulanate, meropenem, doripenem, levofloxacin, and ciprofloxacin; intermediately susceptible to cefoperazone/sulbactam, aztreonam, and norfloxacin; and fully susceptible to piperacillin, piperacillin/tazobactam, ceftazidime, cefepime, gentamicin, tobramycin, and amikacin. A key difference was observed with imipenem: SCPA07 exhibited resistance, whereas SCPA08 showed intermediate susceptibility (Table 1).

**Table 1:** MICs of antimicrobial agents against clinical isolates SCPA07 and SCPA08

Agent	MIC (µg/mL)^a^	
	SCPA07	SCPA08
PIP	16 (S)	16 (S)
TZP	16 (S)	16 (S)
TIC-CLV	≥ 128 (R)	≥ 128 (R)
CPZ-SUL	32 (I)	32 (I)
CAZ	4 (S)	4 (S)
FEP	8 (S)	8 (S)
ATM	16 (I)	16 (I)
IPM	≥ 16 (R)	4 (I)
MEM	≥ 16 (R)	≥ 16 (R)
DOR	≥ 8 (R)	≥ 8 (R)
GEN	≤ 1 (S)	≤ 1 (S)
TOB	≤ 1 (S)	≤ 1 (S)
AMK	≤ 2 (S)	≤ 2 (S)
NOR	8 (I)	8 (I)
LEV	≥ 8 (R)	≥ 8 (R)
CIP	≥ 4 (R)	≥ 4 (R)

*PIP* Piperacillin, *TZP* Piperacillin-tazobactam, *TIC-CLV* Ticarcillin-clavulanate, *CPZ-SUL* Cefoperazone-sulbactam, *CAZ* Ceftazidime, *FEP* Cefepime, *ATM* aztreonam, *IPM* Imipenem, *MEM* Meropenem, *DOR* Doripenem, *GEN* Gentamicin, *TOB* Tobramycin, *AMK* Amikacin, *NOR* Norfloxacin, *LEV* levofloxacin, *CIP* Ciprofloxacin

^a^*S* Susceptible, *I* Intermediate susceptibility, *R* Resistant

### SCPA07 and SCPA08 are isogenic*exoS*^+^/*exoU*^+^strains

Genome sequences of SCPA07 and SCPA08 were obtained using Illumina HiSeq 2000 Sequencer. Both strains were identified as *P. aeruginosa* based on the draft genome using the ANI Calculator [19]. According to the results of genome sequences, SCPA07 and SCPA08 were assigned to the novel ST5159 (*acsA*_32, *aroE*_4, *guaA*_57, *mutL*_5, *nuoD*_1, *ppsA*_15, *trpE*_25). Virulence-associated genes were explored, with both strains possessing 230 genes encoding several known virulence factors, including adherence, antimicrobial activity, antiphagocytosis, biosurfactant, enzyme, iron uptake, protease, quorum sensing, regulation, secretion system, and toxin, as shown in Table S1. Notably, the detection of *exoS*,* exoT*,* exoU*, and *exoY* genes that encoded four key cytotoxins of the type III secretion system of *P. aeruginosa* revealed that SCPA07 and SCPA08 are *exoS*^+^/*exoU*^+^ strains, as they co-carried both *exoU* and *exoS* genes [36]. To elucidate the phylogenetic relationships, a phylogenetic tree was constructed based on the core genome SNPs of SCPA07 and SCPA08 (described in this study), reference strains PAO1 and PA14, alongside 42 *exoS*^+^/*exoU*^+^ *P. aeruginosa* strains with complete genomes available from the NCBI database (Table S2). Generally, two distinct *P. aeruginosa* groups clearly emerged from the tree (Fig. 1B). Group 1 consists of the majority of strains, including PAO1 and all the ST463 strains. SCPA07, SCPA08, the other three ST5159 strains, and an ST4312 strain clustered phylogenetically with PA14 to form group 2. This finding revealed that SCPA07 and SCPA08 are genetically related to PA14 and have evolved independently of the domestically prevalent ST463 (*exoS*^+^/*exoU*^+^) clonal lineage.

Strain SCPA07 was further subjected to whole genome sequencing using Nanopore sequencing, which showed that SCPA07 had a 6,425,849-bp circular chromosome, with an average GC content of 66.44%. Sequence analysis revealed that SCPA07 contained the *P. aeruginosa* pathogenicity island-2 (PAPI-2), which harbors the *exoU* gene [37]. The PAPI-2 of SCPA07, with a length of 20,955 bp, shares 99.78% nucleotide sequence identity with 43% coverage to that of *P. aeruginosa* PA14 (10612 bp). Figure 1C illustrates the organization of the PAPI-2, which showed that the integrase gene *xerC*, *exoU* and its chaperone gene *spcU*, as well as some mobile genetic elements were conserved between SCPA07 and PA14. However, in contrast to that of PA14, where several genes associated with plasmid maintenance and transmission have been deleted in this region [38], SCPA07 has retained these genes. In addition, it has been proposed that the *exoS* gene predated acquisition of *exoU* in group B *P. aeruginosa* strains and was lost due to a targeted deletion event [5]. However, the *exoS* gene was retained in SCPA07 in our study. These findings indicate that SCPA07 appears to be a version of PA14-like *P. aeruginosa* prior to the loss of the *exoS* gene. The coexisting of *exoS* and *exoU* genes confers great virulence potential on SCPA07 and SCPA08 in establishing chronic infections.

Furthermore, WGS analysis revealed that SCPA07 and SCPA08 exhibited high homology, differing by only 10 genetic mutations (Table 2). Of these mutations, 8 were predicted to impact the function of their encoded proteins, which are involved in key biological processes including: O-antigen biosynthesis (*GM001827*, *wzz*), iron metabolism (*irtB*), transcriptional regulator (*ttgR*), cell wall recycling and metabolism (*mpl*), outer membrane transport (*oprD*), and general metabolism (*GM004358*, *GM004987*).

**Table 2:** Genetic mutations of strain SCPA08 relative to SCPA07

Gene / Locus Tag	Nucleotide Mutation (Position)	Putative Function
*ttgR*	+A (coding, 484/633 nt)	HTH type transcriptional regulator TtgR
*oprD*	Δ13 bp (coding, 263–275/351 nt); (CGCCGCC)_2_→_1_ (coding, 943–949/960 nt)	Porin D
*mpl*	+CGGCGCTGGCCG (coding, 1339/1344 nt)	UDP-N-acetylmuramate L-alanyl-γ-D-glutamyl-meso-2,6-diaminoheptandioate ligase
*GM004358*	(TCTATTCA)_3_→_4_ (coding, 2403/2409 nt)	AAA family ATPase
*GM004987*	Δ1 bp (coding, 1317/1563 nt)	Aminoglycoside phosphotransferase domain-containing protein
*GM001827*	G→A; A146V (GCA→GTA)	O-antigen ligase
*wzz*	(G)_5_→_4_ (coding, 93/825 nt)	O-antigen chain length regulator
*irtB*	+G (coding,747/1725 nt)	Iron import permease IrtB
*(glpE / GM000596)*	G→C (intergenic, + 211/−68)^1^	Thiosulfate sulfurtransferase GlpE/hypothetical protein
*tetC*	+AGCAGCGAGGC (coding, 360/591 nt)	Transposon Tn10 TetC protein

For all other mutations, positions are relative to the start codon of the respective gene

¹Position is relative to the start codon of downstream gene (*GM000596*) and stop codon of upstream gene (*glpE*)

### Carbapenem-non-susceptibility is associated with mutations of OprD, elevated expression of efflux pump, and AmpC overproduction

**Fig. 2:**
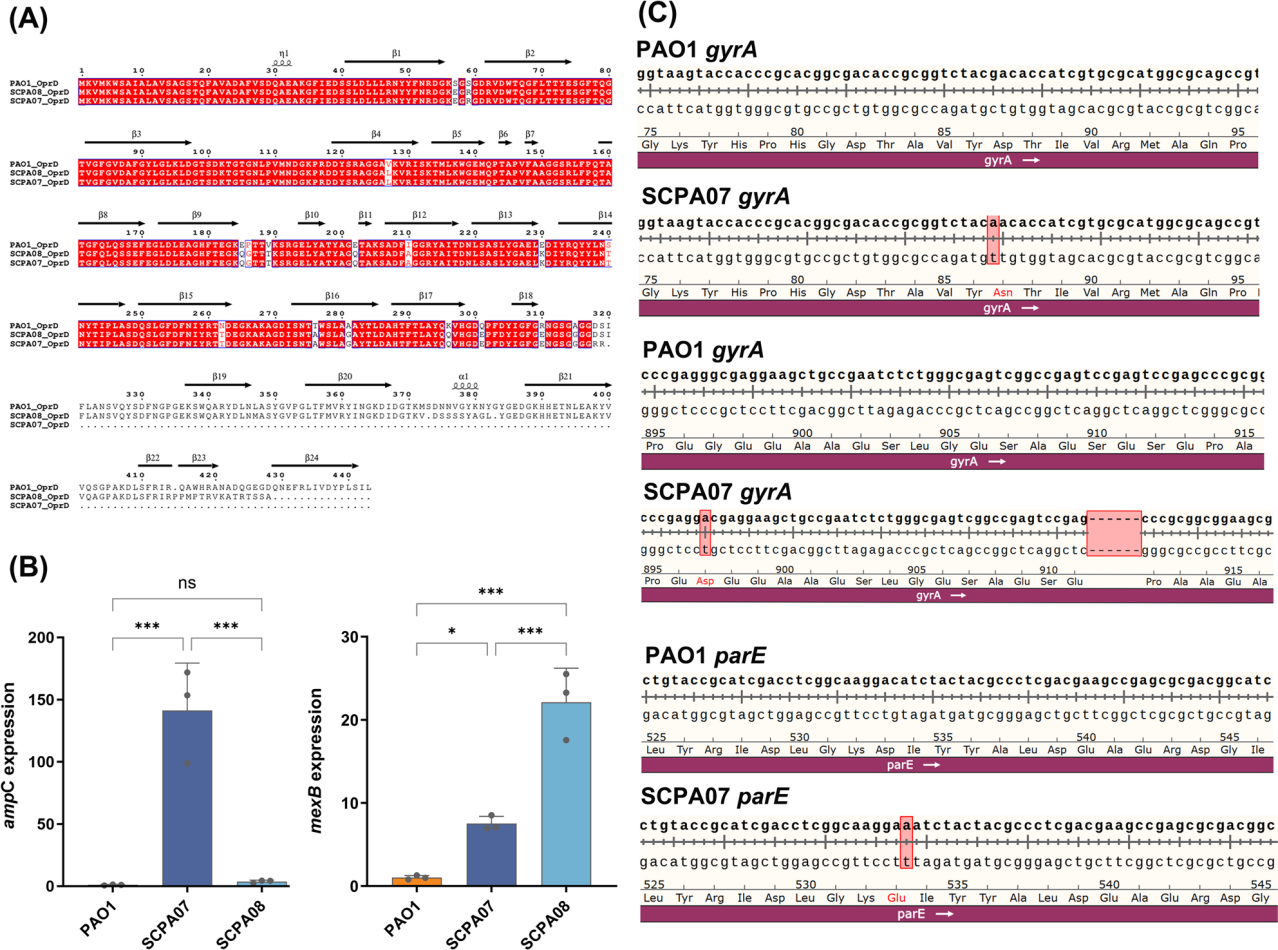
Characterization of resistance mechanisms in clinical *P. aeruginosa* isolates. **A** OprD porin amino acid sequence alignment. OprD sequences from isolates SCPA07 and SCPA08 were compared with reference strain PAO1. Predicted secondary structure of PAO1 OprD porin (SWISS-MODEL) is shown (α-helices, coils; β-strands, arrows). Identical and homologous residues are highlighted on red background and red font, respectively. **B** Expression of *ampC* and *mexB*. Relative mRNA levels in indicated strains. Data are mean ± SD of three independent experiments (triplicates). Significance was assessed by one-way ANOVA with Tukey’s test (*P < 0.05; ***P < 0.001; ns, not significant). **C** Mutations in *gyrA* and *parE* of isolate SCPA07. Sequence alignment with PAO1 reveals nonsynonymous mutations Asp87Asn and Gly897Asp, plus a 6-nt deletion (nt 2734–2739) in *gyrA*, and a single nonsynonymous mutation Asp533Glu in *parE*. Mutant nucleotides are red-boxed, with the deletion marked by a dashed red box; corresponding amino acid changes are red-shaded. Nucleotide numbering and gene directions are indicated

SCPA07 and SCPA08 had identical ARG profiles, mediating resistance to aminoglycosides (*aph(3’)-IIb*), β-lactams (*bla*_OXA−395_ and *bla*_PAO_), fosfomycin (*fosA*), and chloramphenicol (*catB7*). Both strains were resistant to meropenem and non-susceptible to imipenem, yet no carbapenemase genes were detected. *P. aeruginosa* OprD is a specific porin protein that facilitates the uptake of carbapenem molecules across the outer membrane, and mutational inactivation of OprD is a key factor contributing to carbapenem resistance [39, 40]. To examinate *oprD* mutations, we compared the amino acid sequences of OprD from SCPA07 and SCPA08 with that of the PAO1 strain, which revealed several amino acid substitutions (Fig. 2A). Especially, a premature stop codon by point mutation at position 320 (Ile 320*) of OprD was detected in SCPA07, leading the loss of C-terminal region. This finding explains its meropenem resistance, as C-terminal portion of OprD, in particular, loop L7, was responsible for optimal penetration of meropenem and increased its activity [41]. Regarding SCPA08, meropenem resistance was most likely attributed to several amino acid changes in loop L7 region and may also be associated with the premature stop codon at position 429 (Gln429*) of the OprD protein (Fig. 2A). Previous studies revealed that both loops 2 and 3 have a role in imipenem binding to the OprD channel and their mutations could result in imipenem resistance [40, 42]. However, compared with loop 2 or 3 of the OprD protein in PAO1, no amino acid changes were detected in the corresponding regions of SCPA07 and SCPA08, suggesting that other resistance mechanisms against imipenem may be involved.

It has been established that overproduction of efflux pumps and chromosomal cephalosporinase AmpC are major causes of reduced carbapenem susceptibility [39, 43]. Therefore, expression levels of *ampC* and multidrug efflux gene *mexB* in SCPA07 and SCPA08 are depicted by qRT-PCR (Fig. 2B). Results showed that compared to the corresponding level in PAO1, the expression of *mexB* was significantly elevated in both SCPA07 and SCPA08 (*P* = 0.0387 and *P* = 0.0001, respectively). In contrast, *ampC* was overexpressed (at least ten-fold higher expression) in SCPA07 (*P* = 0.0006), but not in SCPA08. The imipenem resistance of *P. aeruginosa* SCPA07 can be possibly explained by the combined effect of *ampC* and *mexB* overproduction, as well as *oprD* mutations. Previous studies have demonstrated that the *mpl* gene is involved in AmpC overproduction [44, 45]. Consistently, sequence alignment revealed a Met297Val missense mutation in the *mpl* gene of SCPA07 relative to that of the reference strain PAO1, which most likely drives the constitutive *ampC* overexpression in this strain. In contrast, a 12 bp insertion mutation in the *mpl* gene of SCPA08 is likely to abrogate the AmpC overproduction phenotype induced by the Met297Val mutation (Table 2).

No acquired quinolone resistance genes were detected in SCPA07 and SCPA08, yet antimicrobial susceptibility testing revealed both strains to be resistant to ofloxacin and ciprofloxacin. It has been shown that the acquisition of chromosomal mutations in the quinolone resistance-determining region of *gyrA*, *gyrB*, *parC*, and *parE* genes is a key mechanism of fluoroquinolone resistance [46, 47]. These genes encode the GyrA/GyrB subunits of DNA gyrase (*gyrA*/*gyrB*) and the ParC/ParE subunits of topoisomerase IV (*parC*/*parE*), respectively. Sequence analysis identified mutations leading to amino acid changes in GyrA (Asp87Asn, Gly897Asp) and ParE (Asp533Glu) in SCPA07 and SCPA08 (Fig. 2C). Also, a 6-nucleotide deletion was identified at nucleotides 2734–2739 of *gyrA*. Besides, elevated efflux pump production, such as MexAB-OprD, may also contribute to quinolone resistance in both strains [48].

### SCPA07 and SCPA08 are both ‘hypermutator’ strains with divergent growth and phenotypic traits

Clinical isolates with mutations in genes involved in DNA repair, such as *mutS* and *mutL*, frequently show a hypermutator phenotype, which could result in faster accumulation of adaptive mutations during infection [11]. Amino acid sequence analysis revealed missense mutations in both *mutS* (Ala772Thr) and *mutL* (Ala391Val), indicating that both SCPA07 and SCPA08 could be hypermutator strains. Mutations in pathoadaptive genes are considered the main drivers of pathogen fitness [49]. A previous study by Marvig et al. identified 52 candidate ‘pathoadaptive genes’ targeted by mutations to optimize pathogen fitness [49]. We found at least one nonsynonymous mutation in 35 out of the 50 candidate pathoadaptive genes (two genes were not found) in SCPA07 (Table S4). The functional class analysis revealed that these mutated genes are mainly involved in motility and attachment, antibiotic resistance and susceptibility, transcriptional regulators, and other aspects.

**Fig. 3:**
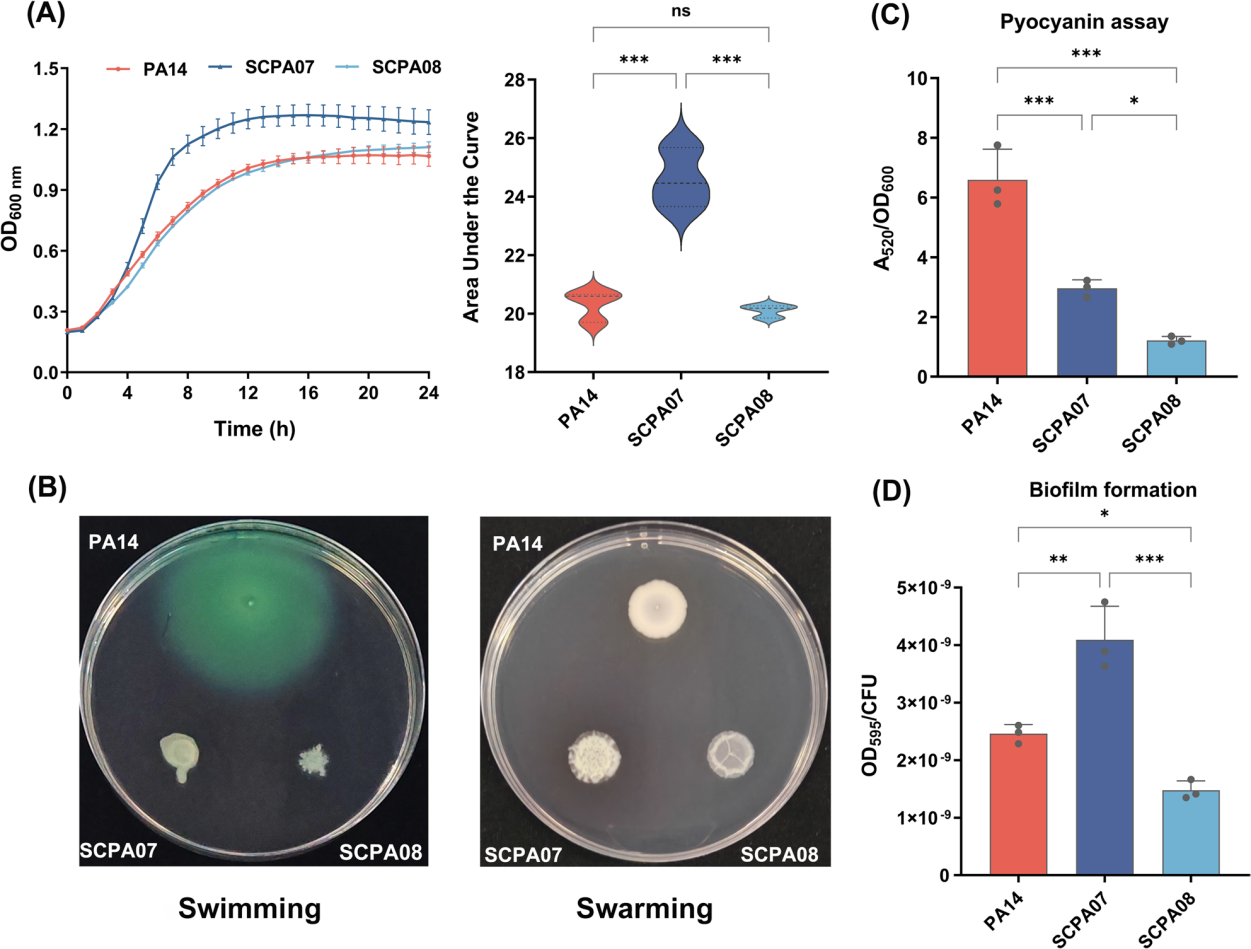
Virulence-associated characteristics of *P. aeruginosa* SCPA07 and SCPA08. **A** Growth assay. Growth curves were monitored by OD₆₀₀ measurement over 24 h, and converted into AUC to evaluate growth ability. **B** Representative images of swimming and swarming motility. **C** Quantitation of pyocyanin. **D** Crystal violet staining for quantification of biofilm formation. Data are presented as the mean ± SD from at least three independent experiments performed in triplicate. Statistical analyses were evaluated using one-way ANOVA followed by Tukey’s multiple comparisons test. *P < 0.05, **P < 0.01, ***P < 0.001; ns, not significant

To investigate the effects of these adaptive mutations on bacterial fitness, the growth curves of SCPA07 and SCPA08 were determined over a 24-hour incubation period in rich LB broth, and differences in the area under AUC among the strains were analyzed (Fig. 3A). Results showed that SCPA07 had a significantly higher AUC compared with SCPA08 and the reference strain PA14 (SCPA07 vs. SCPA08: 24.60 ± 1.017 vs. 20.10 ± 0.2211, *P* = 0.0004; SCPA07 vs. PA14: 24.60 ± 1.017 vs. 20.31 ± 0.5320, *P* = 0.0006), suggesting that SCPA07 possessed superior growth dynamics.

The motility assay demonstrated that both SCPA07 and SCPA08 exhibited significantly impaired swimming motility relative to the reference strain PA14: PA14 formed a large, uniform green halo, SCPA07 developed a relatively compact, rounded colony, and SCPA08 produced a small, dense colony with minimal diffusion from the inoculation site (Fig. 3B). On the swarming plate, SCPA07 formed a colony with a textured, irregular margin and a spreading diameter of 13.14 mm, while SCPA08 produced a more compact colony with a distinct, patterned margin (spreading diameter: 11.28 mm). Both diameters were smaller than that of PA14, whose colony spread to 15.32 mm (Fig. 3B). Overall, SCPA07 showed higher swimming and swarming motility than SCPA08. To determine the genetic basis of the motility phenotype, we compared the sequences of core flagellar genes between SCPA07, SCPA08, and PA14. The majority of flagellar genes, including key regulators such as *fleR* and *fliA*, were highly conserved, with only sporadic synonymous substitutions or conservative amino acid changes. However, we identified a marked divergence in *fliC* gene, which encodes the flagellin subunit and is the major structural component of the flagellar filament [50]. SCPA07 and SCPA08 had multiple non-synonymous mutations and small insertions/deletions in *fliC* compared with PA14 (99 mismatches and 8 indels across the 1467-bp gene). Given that FliC composes the filament backbone, such extensive sequence variation is likely to alter the assembly and stability of the flagella, ultimately affecting the motility of the bacteria.

Pyocyanin is a well-characterized virulence factor produced by 95% of *P. aeruginosa* isolates, and it is a secreted redox-active phenazine toxin that exacerbates infection by inducing oxidative stress and causing host tissue damage [51]. Here, SCPA07 exhibited significantly higher pyocyanin production than SCPA08 (*P* < 0.05), whereas both strains synthesized substantially lower pyocyanin levels relative to the reference strain PA14 (*P* < 0.001, Fig. 3C). Pyocyanin production involves two homologous core loci biosynthetic pathways (*phzA1B1C1D1E1F1G1* and *phzA2B2C2D2E2F2G2*), together with three genes *phzM*,* phzS*, and *phzH* encoding enzymes converting phenazine-1-carboxylic acid to pyocyanin [52]. Sequence analysis of the *phz* operon revealed that the *phzC2*, *phzE2*, and *phzF2* genes were absent in both SCPA07 and SCPA08 (Table S1), a finding that may explain their impaired pyocyanin biosynthesis ability.

Biofilm formation is a key contributing factor to the establishment of persistent, chronic infections, especially in *P. aeruginosa.* The biofilm formation assay, coupled with the bacterial counting method, demonstrated that SCPA07 exhibited the most robust biofilm-forming ability, which was significantly higher than that of SCPA08 (*P* < 0.001) and the reference strain PA14 (*P* < 0.01). In contrast, SCPA08 displayed the weakest biofilm formation, with its biomass being approximately 2.8-fold lower than that of SCPA07 (Fig. 3D). SNP analysis revealed that, in comparison with strain SCPA07, SCPA08 carried two distinct mutations in O-antigen biosynthesis: a nonsynonymous mutation in *GM001827* (encoding an O-antigen ligase) and a frameshift mutation in *wzz* (encoding an O-antigen chain length regulator). A previous study showed that lipopolysaccharide (LPS) -like materials constitute a component of a biofilm matrix in *P. aeruginosa* PA14 [53]. Besides, the O-antigen of LPS has been demonstrated to be tightly correlated with biofilm formation in *Salmonella Enteritidis* and *Actinobacillus pleuropneumoniae* [54, 55]. In addition, the Pel polysaccharide is well recognized as a major structural component of *P. aeruginosa* PA14 biofilms and plays a dominant role in early biofilm development and matrix integrity [56]. Genomic analysis revealed no duplication or copy number variation of the *pel* operon in either SCPA07 or SCPA08 compared with the reference strain PA14. However, six amino acid substitutions were identified in Pel-related proteins between our isolates and PA14, which may contribute to the enhanced biofilm-forming capacity observed in SCPA07 relative to PA14. Moreover, the marked reduction in biofilm formation in SCPA08 compared with SCPA07 might be attributable to the aforementioned O-antigen-related mutations. Collectively, the variations in the Pel protein and the O antigen jointly led to the biofilm phenotypes of these two clonally related isolates.

### SCPA07 and SCPA08 showed divergent virulence and stress tolerance profiles.

**Fig. 4:**
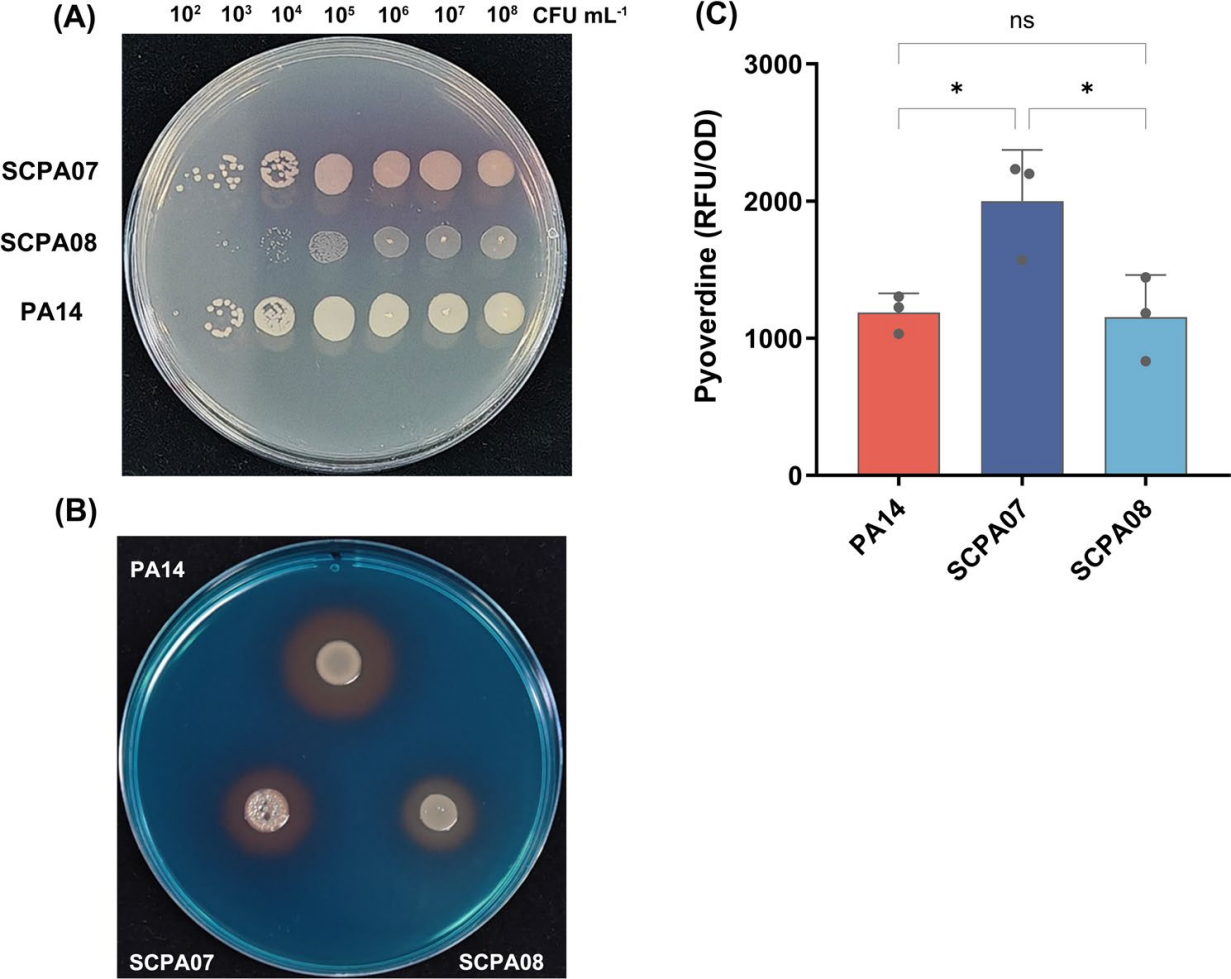
Comparative analysis of iron acquisition capabilities in *P. aeruginosa* clinical isolates. **A** Growth under iron-limited conditions. Serial dilutions (10^8^ to 10^2^ CFU/mL) of logarithmic-phase cultures were spotted onto LB agar containing the iron chelator 2,2′-dipyridyl (300 µM) and incubated overnight at 37 °C. **B** Siderophore production on a CAS-LB agar plate. Cultures were spotted onto the plate and incubated at 37 °C for 48 h. Formation of an orange-yellow halo around each bacterial spot indicates siderophore secretion. **C** Quantification of pyoverdine. Fluorescence of culture supernatants was measured (excitation 400 nm, emission 460 nm) and normalized to cell density (OD₆₀₀), expressed as relative fluorescence units per OD (RFU/OD). Data are presented as the mean ± SD from at least three independent experiments in triplicate. Statistical analysis was performed using one-way ANOVA followed by Tukey’s multiple comparisons test (*P < 0.05; ns, not significant)

Iron is essential for bacterial growth and virulence. Under normal in vivo conditions, iron is not readily available due to its low solubility and the activity of host iron-binding proteins [51]. On the iron-limited LB plate, SCPA07 displayed growth ability comparable to that of PA14: an obvious bacterial lawn formed at high concentrations, and colonies could still develop even at concentrations as low as 10^2^. In contrast, SCPA08 exhibited significantly poorer growth across all concentration gradients (Fig. 4A). This result reveals that SCPA07 exhibits a distinct growth advantage under iron-limited conditions compared with SCPA08.

In the iron-limited host environment, *P. aeruginosa* needs to retrieve iron from the host in order to infect and multiply within tissues or body fluids [57]. To fulfill iron requirements, siderophore production represents a primary strategy utilized by *P. aeruginosa.* According to the halo formation on the CAS-LB agar plates, our results demonstrated that SCPA07 exhibited a higher level of siderophore production than strain SCPA08 (Fig. 4B). Whereas, both strains displayed lower siderophore secretion capacities relative to PA14. Pyoverdine has a peptide nature and is considered the major siderophore that acquires iron from the surrounding medium [58]. The pyoverdine production assay showed that strain SCPA07 produced significantly higher levels of pyoverdine, and the latter two strains (SCPA08 and PA14) exhibited comparable pyoverdine levels (Fig. 4C). Collectively, these results indicate that SCPA07 has greater siderophore synthesis capacity than SCPA08, which explains its superior growth under iron-limited conditions. SNP analysis showed that, compared to SCPA07, a G base was inserted at position 747 of the *irtB* gene (encoding an iron import ATP binding/permease protein) in SCPA08. IrtB was shown to be involved in export and import of siderophores across the membrane and the consequent iron uptake in *Mycobacterium tuberculosis* [59]. Therefore, the reduction in iron uptake in SCPA08 is most likely attributed to the frameshift mutation of *irtB*.

**Fig. 5:**
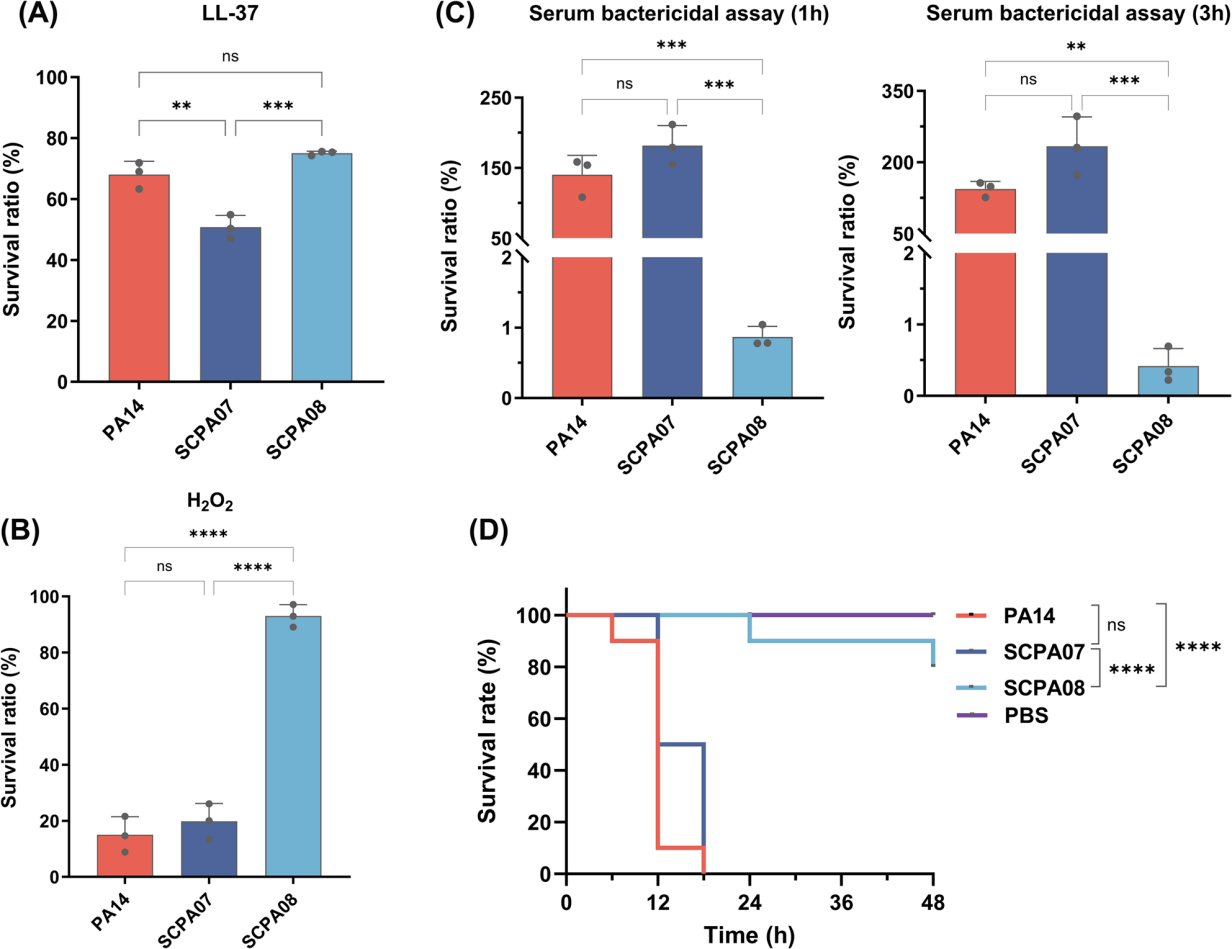
Tolerance to host defense factors and in vivo virulence of *P. aeruginosa* strains. **A**–**C** Tolerance to host defense factors. Logarithmic-phase bacteria were treated with (**A**) LL-37, (**B**) H_2_O_2_, or (**C**) human serum (for 1–3 h). Treatments with PBS or ddH_2_O served as controls. Survival was determined by quantifying CFU after each treatment. ** D ***G. mellonella* infection model. Larvae were injected with 10 µL of bacterial suspension (5 × 10^3^ CFU per larva) or PBS (negative control) and monitored for survival over 48 h. Data represent the mean ± SD from three independent experiments performed in triplicate. Statistical significance was evaluated using one-way ANOVA (**A**–**C**) or the log-rank (Kaplan–Meier) test (**D**) (*P < 0.05, **P < 0.01, ***P < 0.001, ****P < 0.0001; ns, not significant)

To evaluate the fitness of SCPA07 and SCPA08 under host-derived stress conditions, we assayed their survival capacity against three host antimicrobial effectors, namely human host defense peptide LL-37, H_2_O_2_, and human serum. In the presence of 50 µg/mL LL-37, only 50.77 ± 3.873% SCPA07 survived following a 1-hour incubation period, significantly lower than that of SCPA08 (75.08 ± 0.6591%, *P* = 0.0003) (Fig. 5A). In the presence of 50 mM H_2_O_2_, approximately 19.81 ± 6.367% of SCPA07 was survived following a 20-minute treatment, whereas SCPA08 exhibited significant resistance to H_2_O_2_-mediated killing, with a survival rate of (93.04 ± 4.020) % (*P* < 0.0001) (Fig. 5B). These results indicate that SCPA08 exhibits a significant growth advantage under stress conditions of LL-37 and H_2_O_2_.

Under 75% serum exposure, SCPA07 displayed robust resistance to the killing and even propagated in this hostile environment, with survival rates of 181.9 ± 28.51% at 1 h and 233.9 ± 61.52% at 3 h. In contrast, SCPA08 showed marked susceptibility to serum-mediated killing, with its survival rate dropping to 0.8680 ± 0.1528% at 1 h and further declining to 0.4175 ± 0.2451% over the subsequent 2-hour period. The survival rate of SCPA08 was significantly lower than that of SCPA07 at the corresponding time point (*P* = 0.0002 at 1 h, and *P* = 0.0006 at 3 h) (Fig. 5C). These findings suggest that SCPA08 is more vulnerable in the serum than SCPA07. In addition, compared with the reference strain PA14, SCPA07 exhibited significantly reduced resistance to LL-37 (*P* = 0.0019), while SCPA08 demonstrated diminished resistance to serum killing (*P* = 0.0007) but markedly enhanced tolerance to H_2_O_2_ (*P* < 0.0001) (Fig. 5A-C).

The virulence of SCPA07 and SCPA08 was assessed using the *G. mellonella* infection model. Each larva was injected with 5*10^3^ CFU SCPA07, SCPA08 or the reference strain PA14 and monitored mortality over 48 h. At 6 h post-infection (p.i.), 10% of the PA14-infected larvae succumbed to infection; the mortality rate subsequently increased to 90% by 12 h p.i., and all larvae died by 18 h p.i. In comparison, none of the larvae infected with SCPA07 died at 6 h p.i.; the mortality rate had reached 50% at 12 h p.i., and all larval also died by 18 h p.i. (Fig. 5D) Statistical analysis revealed no statistically significant difference in virulence between these two strains, indicating that SCPA07, like PA14, is also a highly virulent strain. In contrast, SCPA08 exhibited significantly attenuated virulence compared to SCPA07 (*P* < 0.0001, Log-rank (Mantel-Cox) test), as it induced merely 10% mortality rate at 24 h p.i., and a 20% mortality rate by 48 h p.i.

## Discussion

In this study, two homologous carbapenem-non-susceptible *P. aeruginosa* isolates SCPA07 and SCPA08 were isolated from the same BALF sample in a patient with bacterial pneumonia. Genomic analysis demonstrated that these two strains are closely related to PA14 and harbor both *exoS* and *exoU* genes, yet they are phylogenetically distinct from the *exoS*⁺/*exoU*⁺ ST463 strains that predominate in clinical settings across China. ExoU is known as the most virulent T3SS effector and secretion of the toxin ExoU is a marker for highly virulent *P. aeruginosa* isolates from patients with hospital-acquired pneumonia [60]. ExoS can promote the survival and dissemination of *P. aeruginosa* by inhibiting host immune defense and disrupting tissue barriers [61, 62]. The co-expression of ExoU and ExoS in SCPA07 and SCPA08 could induce more severe infections and higher mortality [8].

The within-host mutation rate is a key factor governing the potential for bacterial pathogens to undergo genetic adaptation to the host environment. In parallel, the generation of mutations is frequently accelerated in clinical *P. aeruginosa* strains, which were referred to as “hypermutators” [63]. Here, owing to the occurrence of missense mutations in the *mutS* and *mutL* DNA repair genes, both SCPA07 and SCPA08 were identified as hypermutators. They carried mutations across a broad range of pathoadaptive genes, suggesting an enhanced pathogenic potential that enables rapid, adaptive responses to the changing host environment.

A single *P. aeruginosa* strain frequently evolves into genetically distinct but clonally related subpopulations over the course of an infection; such divergence arises from differential selective pressures and genetic drift acting on subpopulations in different lung regions [64]. In this study, we infer that SCPA07 and SCPA08 may originate from distinct regions of the lung, given that they exhibit marked phenotypic divergence and were isolated from BALF—a sample type that typically spans multiple anatomical areas of the lung. Among these two strains, the most prominent characteristics of SCPA07 include a rapid growth rate in rich medium, robust biofilm formation ability, high virulence, as well as a growth advantage under iron-limiting conditions and serum-mediated killing stress. In contrast, its variant SCPA08 exhibited slow growth, poor motility, reduced pyocyanin production, loss of acute virulence, yet enhanced resistance to the host antimicrobials LL-37 and H_2_O_2_. It has been shown that modulating LPS synthesis and structure represents a characteristic adaptive change of *P. aeruginosa* during chronic infection, which facilitates host immune evasion, enhanced antimicrobial resistance, and persistent colonization [65]. Here, relative to SCPA07, we identified a frameshift mutation in the LPS O-antigen chain length regulator *wzz* in SCPA08, which is highly likely to induce unregulated extension of the O-antigen chain along with elevated synthetic levels [66]. We propose that the changes in O-antigen may lead to slower bacterial growth, increased serum susceptibility, and consequently marked reduction in the in vivo virulence of SCPA08 [67]; conversely, this O-antigen alteration may confer the strain with elevated resistance to antimicrobial peptides LL-37 [68].

During chronic lung infections, *P. aeruginosa* adapts to the host environment by evolving toward a state of reduced bacterial invasiveness, which promotes bacterial persistence while avoiding overwhelming host injury [11]. In this study, SCPA08 is predicted to facilitate persistent colonization in the lung via reduced growth rate and enhanced resistance to host antimicrobials. In contrast, the highly virulent variant SCPA07 could be responsible for the proliferation and dissemination of the bacterial population as well as host tissue damage in the pulmonary niche. Such division of labor and synergistic cooperation jointly sustain the viability of the bacterial community. Given that SCPA07 grows fast and is able to resist serum-mediated killing, it is associated with an increased risk of developing bacteremia. Furthermore, the robust biofilm-forming capability also renders SCPA07 a persistent and hard-to-eradicate pathogen.

## Conclusions

Collectively, our study characterized two mixed-variants of *P. aeruginosa* co-colonizing the lung of the same patient and demonstrated their adaptive evolutionary advantages acquired via genetic mutations. Since this bacterial population collectively exhibits high drug resistance, robust persistence, and high virulence, its thorough elimination is highly challenging in clinical settings. The deployment of novel alternative therapies, such as bacteriophage therapy and iron-chelating agents, or combined regimens of antibiotics and alternative therapies is warranted to combat this clinically significant pathogen.

## Supplementary Information

Supplementary Material 1.

Supplementary Material 2.

## Data Availability

Whole-genome sequences of strains SCPA07 and SCPA08 have been deposited into GenBank under the accession no. JBUEHF000000000 and JBUEHG000000000, respectively. All other relevant data generated or analyzed during this study are included in this published article and its supplementary information files, or are available from the corresponding author on reasonable request.

## Abbreviations

*P. aeruginosa*: *Pseudomonas aeruginosa*
CF: Cystic fibrosis
BALF: Bronchoalveolar lavage fluid
WGS: Whole-genome sequencing
qRT-PCR: Quantitative reverse-transcription PCR
CLSI: Laboratory Standards Institute
LB: Luria-Bertani
ANI: Average nucleotide identity
ARGs: Antibiotic resistance genes
VFDB: Virulence Factor Database
SNP: Single-nucleotide polymorphism
AUC: Area under the curve
CFU: Colony-forming units
RFU: Relative fluorescence units
CAS: Chrome azurol S
CTAB: Cetyltrimethylammonium bromide
*Galleria mellonella*: *G. mellonella*
NCBI: National Center for Biotechnology Information
PAPI-2: Pathogenicity island-2
LPS: Lipopolysaccharide
