# Supplementary material for: Characterization of two exoU+/exoS+ carbapenem-non-susceptible Pseudomonas aeruginosa co-colonizing the lung of a bacterial pneumonia patient
Source: BMC Microbiol. 2026 May 18;26:615. doi: 10.1186/s12866-026-05172-8 (PMC13352818; doi:10.1186/s12866-026-05172-8)
Supplement: Supplementary file 2 — Supplementary Material 2. [file 12866_2026_5172_MOESM2_ESM.docx]

**Table S2 Detailed information of *P. aeruginosa* strains used in the phylogenetic tree.**

| **Name** | **Source** | **Locations** | **Year** | **Accession no.** |
| --- | --- | --- | --- | --- |
| R20-48 | Not specified | China | 2023 | CP138391 |
| TL3773 | blood | China | 2018 | CP080011 |
| SRRSH1002 | Not specified | China | 2018 | CP064397 |
| SRMPA1728 | Not specified | China | 2024 | CP192438 |
| AS02 | bronchoalveolar lavage fluid | China | 2019 | CP187730 |
| AS01 | bronchoalveolar lavage fluid | China | 2019 | CP187732 |
| SRRSH1408 | Not specified | China | 2018 | CP064395 |
| SRRSH1101 | Not specified | China | 2018 | CP064393 |
| PA328 | ascites | China | 2023 | CP185402 |
| ZY94 | sputum | China | 2021 | CP125361 |
| ZY36 | sputum | China | 2022 | CP125365 |
| ZY1710 | sputum | China | 2022 | CP125367 |
| ZY156 | urine | China | 2021 | CP125363 |
| R18-20 | sputum | China | 2023 | CP136014 |
| PA2207 | feces | China | 2021 | CP080289 |
| PA64 | sputum | China | 2022 | CP159840 |
| P23 | Not specified | China | 2019 | CP065417 |
| PA30 | Not specified | China | 2021 | CP102441 |
| PA3117 | Not specified | China | 2022 | CP159841 |
| ZYPA1927 | sputum | China | 2023 | CP133751 |
| QZPH16 | Not specified | China | 2017 | CP078004 |
| SRRSH2790 | Not specified | China | 2017 | CP077994 |
| QZPH21 | Not specified | China | 2017 | CP078002 |
| R31 | sputum | China | 2015 | CP061850 |
| PAE3 | sputum | China | 2024 | CP154349 |
| NDTH7329 | Not specified | China | 2018 | CP078006 |
| HS18-89 | urine | China | 2018 | CP084321 |
| NDTH9845 | Not specified | China | 2018 | CP073080 |
| PA12 | Not specified | China | 2019 | CP136598 |
| NDTH10366 | Not specified | China | 2020 | CP064401 |
| SRRSH1120 | Not specified | China | 2018 | CP077999 |
| P33 | Not specified | China | 2019 | CP065412 |
| R13-28 | Not specified | China | 2023 | CP138389 |
| S11-17 | Not specified | China | 2023 | CP138578 |
| S1 | soil | China | 2023 | CP148035 |
| E113 | oropharynx, sputum, or bronchoalveolar lavage | USA | 2010 | CP093354 |
| Pa150 | Not specified | China | 2018 | CP094677 |
| M28A1 | cow manure | China | 2004 | CP015649 |
| JT86 | soil | China | 2018 | CP062219 |
| PAO1 | wound | Australia | 2006 | AE004091 |
| NMI18803 | Not specified | China | 2000 | CP128275 |
| PATH-33 | urine | Thailand | 2022 | CP191503 |
| PATH-12 | Pus | Thailand | 2021 | CP191509 |
| SCAIDPHRX1-2019 | Pharynx | Kazakhstan | 2019 | CP053686 |
| 80664 | ear canal swab (Canis lupus familiaris) | UK | 2024 | CP173149 |
| PA14 | wound | USA | 2006 | CP000438 |
| SCPA08 | bronchoalveolar lavage fluid | China | 2024 | This study |
| SCPA07 | bronchoalveolar lavage fluid | China | 2024 | This study |
| Rectal 2 P14 | Rectal swab 2 | Ecuador | 2022 | JARNKP010000000 |
| Wound 3 P14 | wound | Ecuador | 2022 | JARNKM010000000 |
| Femur 2 P14 | Femur | Ecuador | 2022 | JARNKN000000000 |

| **Primer** | **5'-sequence-3'** | **Length (bp)** |
| --- | --- | --- |
| *oprD*-F | CCTGCTGCTCCGCAACTA | 18 |
| *oprD*-R | CCACGGTGCCTTGAGTGA | 18 |
| *mexB*-F | CAAGGGCGTCGGTGACTTCCAG | 22 |
| *mexB*-R | ACCTGGGAACCGTCGGGATTGA | 22 |
| *ampC*-F | GCTGGGAAGCCTACGACTGG | 20 |
| *ampC*-R | CCCGCTCGGCATTGGGATAG | 20 |
| *rpsL*-F | GCAAGCGCATGGTCGACAAGA | 21 |
| *rpsL*-R | CGCTGTGCTCTTGCAGGTTGTGA | 23 |

**Table S3 qRT-PCR Primer sequences.**

**Table S4** **Detailed information about mutations in pathoadaptive genes in strain SCPA07**

| **Gene name** | **SCPA07 mutation** | **Product** |
| --- | --- | --- |
| *gyrB* | None | DNA gyrase subunit B |
| *htrB* | None | Lipid A biosynthesis lauroyltransferase |
| *yecS* | None | L-cystine transport system permease protein YecS |
| *mexR* | V126E | Multidrug resistance operon repressor |
| *mexA* | A17S | Multidrug resistance protein MexA |
| *mexB* | I186V | Multidrug resistance protein MexB |
| *algU* | None | RNA polymerase sigma-H factor |
| *mucA* | None | Sigma factor AlgU negative regulatory protein |
| *rbdA* | V804L, S811P | hypothetical protein |
| *oprD* | Multiple mutations¹ | Porin D |
| *PA0977* | Not found | hypothetical protein |
| *lasR* | None | Transcriptional activator protein LasR |
| *PA1471* | Not found | hypothetical protein |
| *dnaX* | Δ460-475; V366A, T432A, V477A, T532A,G574A | hypothetical protein |
| *PA1677* | R40S, S61N, E196R | Streptothricin hydrolase |
| *mexZ* | D83E, L38R, | HTH-type transcriptional regulator TtgR |
| *pcoA* | A63T, V401M, N402K, S415N; Δ424-441 | Copper resistance protein A |
| *PA2099* | Q44E, A48T, H50Q, H106Q, Q189R, H212Q | hypothetical protein |
| *pvdS* | None | hypothetical protein |
| *PA2455* | G25E | hypothetical protein |
| *PA2490* | T48S, R114C | hypothetical protein |
| *mexS* | D249N | L-threonine 3-dehydrogenase |
| *PA2602* | A32T, H35P, H43R, | 3-mercaptopropionate dioxygenase |
| *pelA* | C25R, T41A, H141Y, I438V, T453A, G837A | hypothetical protein |
| *wbpM* | N4R, I7V, G11S, L12M, Y16W, M19L, A24T, V26I, T27L, L38V, E44D,Δ48-49, Δ51-52, A59T, L62V, V63I,L103V, S109G, P110A, V113P | UDP-N-acetyl-alpha-D-glucosamine C6 dehydratase |
| *gyrA* | D87N, G887D, Δ909-910 | DNA gyrase subunit A |
| *PA3222* | P62S, S196G, I271V, G284N, K286P, T287V | hypothetical protein |
| *PA3290* | L172M, V173I, E175N,S177A, W180G, T181V, V182A, S183K,T184V, N217D, I231V, A243T,V254A,D256G, L311Q, S312A, P316S,V333I,M352L,K366Q,W367G,E414G,A423K,P424S,E433V,V436E,V438T,K440E,I441T,N492S,P863L,K873Q | hypothetical protein |
| *ykoM* | None | hypothetical protein |
| *nalD* | None | HTH-type transcriptional repressor BepR |
| *wspE* | L128Q, V156A, V420A, A477T | Sensor histidine kinase RcsC |
| *wspA* | T72V, Q120L, A121G, D125A, E159D, M167L, D180E | hypothetical protein |
| *PA3939* | A4P, T13A, V37A, E47D, A67V, V103I, R125P, Y183C, L188V, C204R, A323T, E281A | putative FMNH2-dependent monooxygenase SfnC |
| *mpl* | M297V | UDP-N-acetylmuramate--L-alanyl-gamma-D-glutamyl-me... |
| *phzB1* | Q154E | Phenazine biosynthesis protein PhzB1 |
| *PA4311* | S44G | GDP-mannose-dependent alpha-(1-6)-phosphatidylinos... |
| *bifA* | None | hypothetical protein |
| *pilD* | T18A | Type 4 prepilin-like proteins leader peptide-proce... |
| *nfxB* | None | hypothetical protein |
| *morA* | R53G, D495E | hypothetical protein |
| *PA4642* | None | hypothetical protein |
| *retS* | A46V, I324V, | Sensor histidine kinase RcsC |
| *PA4963* | Q162R, V213A | hypothetical protein |
| *aceE* | None | Pyruvate dehydrogenase E1 component |
| *aceF* | K98Q, D445E | Dihydrolipoyllysine-residue acetyltransferase comp... |
| *pilQ* | A106T, A135T, T266M | Type IV pilus biogenesis and competence protein PilQ |
| *phaF* | S121G, K128R, T186P, T204A, T213A, | hypothetical protein |
| *PA5177* | None | GMP/IMP nucleotidase YrfG |
| *vgrG* | N124D | Actin cross-linking toxin VgrG1 |
| *betT* | M1V, E32A, A299T, P514H | Osmo-independent choline transporter BetT1 |
| *cmpR* | None | HTH-type transcriptional activator CmpR |
| *pdxY* | R50Q, T248A | Pyridoxal kinase PdxY |

*Footnote: ¹ See Figure 2A for detailed amino acid substitutions.
